# Increased co-expression of genes harboring the damaging *de novo* mutations in Chinese schizophrenic patients during prenatal development

**DOI:** 10.1038/srep18209

**Published:** 2015-12-15

**Authors:** Qiang Wang, Miaoxin Li, Zhenxing Yang, Xun Hu, Hei-Man Wu, Peiyan Ni, Hongyan Ren, Wei Deng, Mingli Li, Xiaohong Ma, Wanjun Guo, Liansheng Zhao, Yingcheng Wang, Bo Xiang, Wei Lei, Pak C Sham, Tao Li

**Affiliations:** 1 Mental Health Center, West China Hospital, Sichuan University, Chengdu, Sichuan, P R China; 2 State Key Laboratory of Biotherapy, Psychiatric laboratory, West China Hospital, Sichuan University, Chengdu, Sichuan, P R China; 3 State Key Laboratory of Brain and Cognitive Sciences, Centre for Genomic Sciences and Department of Psychiatry, University of Hong Kong, Pokfulam, S.A.R. China; 4 Biobank, West China Hospital, Sichuan University, Chengdu, Sichuan, P R China

## Abstract

Schizophrenia is a heritable, heterogeneous common psychiatric disorder. In this study, we evaluated the hypothesis that *de novo* variants (DNVs) contribute to the pathogenesis of schizophrenia. We performed exome sequencing in Chinese patients (N = 45) with schizophrenia and their unaffected parents (N = 90). Forty genes were found to contain DNVs. These genes had enriched transcriptional co-expression profile in prenatal frontal cortex (Bonferroni corrected *p* < 9.1 × 10^−3^), and in prenatal temporal and parietal regions (Bonferroni corrected *p* < 0.03). Also, four prenatal anatomical subregions (VCF, MFC, OFC and ITC) have shown significant enrichment of connectedness in co-expression networks. Moreover, four genes (*LRP1*, *MACF1*, *DICER1* and *ABCA2*) harboring the damaging *de novo* mutations are strongly prioritized as susceptibility genes by multiple evidences. Our findings in Chinese schizophrenic patients indicate the pathogenic role of DNVs, supporting the hypothesis that schizophrenia is a neurodevelopmental disease.

Schizophrenia is a complex clinical syndrome, which is characterized by abnormal perception, thought process, disorganized speech and behavior. It affects about 1% of the population worldwide and is considered as a heritable, heterogeneous common psychiatric disorder^1^. Results from the largest genome-wide association study (GWAS) in 36,989 cases with schizophrenia and 113,075 controls have led to the identification of 108 common loci with small effect (OR < 1.4) for schizophrenia^2^. While confirming some of the key hypotheses on the pathogenesis of schizophrenia, such as neurotransmitter dysfunction, this study also demonstrated the previously unknown roles of biological pathways involved in the development of the disorder. However, it remains unclear as to why all the identified common variants represent only a modest portion of the overall heritability of schizophrenia^3^. One of the possibilities is that a considerable number of rare variants are ‘hidden’ across linkage disequilibrium (LD) blocks, which also contribute to the genetic risk for the disorder^4^. Thus, there is a probability of occurrence of multiple rare variants with minor allele frequency (MAF) < 1%, and the association of each of these with a potential effect on the risk of developing schizophrenia. These effects may accumulate to present the onset of schizophrenia.

To date, various *de novo* variants (DNVs) have been identified as contributors for a number of neurodevelopmental disorders, such as autism spectrum disease (ASD)^5^,^6^, mental retardation^7^, and schizophrenia^8^,^9^,^10^,^11^. The hypothesis of ‘common disease, rare variants’ is increasingly becoming as appealing as the ‘common disease, common variants’ hypothesis. However, while the contribution of rare DNVs has been unfolded in western population, it has not been reported in population in eastern countries. Therefore, it is important to explore the pathogenic potential of DNVs and their genomic influence on schizophrenia in ancestrally independent samples. In addition, although schizophrenia is associated with heritability rate of as high as 80%^12^, a large fraction of the population is without a family history of the disease (sporadic cases). Therefore, Xu *et al*. investigated the relationship between copy number variants (CNVs) and family history in schizophrenia. They demonstrated that *de novo* copy number mutations were significantly associated with sporadic rather than familial patients with schizophrenia^13^. Moreover, the study indicated differences in genetic mechanism between patients with sporadic and familial schizophrenia, providing new insights into the role of *de novo* non-synonymous variants in schizophrenia. However, in a larger sample, Fromer *et al*. demonstrated that there was no difference in the rate of *de novo* mutations in patients with sporadic and familial schizophrenia^14^.

Recently, studies have also investigated whether there was a functional correlation between the fetal expression bias of the mutated genes and the neurodevelopmental impact of the corresponding mutations, which focused on genetic control of transcription in the brain areas especially the dorsolateral prefrontal cortex (DLPFC)^8^,^11^ and hippocampus cortex (HIP)^8^ during the stages of neurodevelopment. Although these findings are promising, they have not been replicated in independent sample sets yet. In addition, previous studies have shown that the accrued risk of neurodevelopmental diseases, such as ASD^15^,^16^,^17^,^18^, intellectual disability^19^,^20^, and schizophrenia^21^ was associated with greater paternal age as the rate of DNVs disrupting the gene functions increases. However, it is still equivocal for schizophrenia because of its genetic heterogeneity and complexity of phenotypes. These issues need to be retested in different population as well.

In this study, we conducted the exome sequencing analysis in 45 schizophrenia-parent trios in order to provide evidence for the contribution of DNVs to the pathogenesis of schizophrenia. We assessed the potential pathogenic impact and functional characterization of genes harboring the damaging DNVs, and analyzed co-expression network of these genes in different brain regions at different developmental stages Furthermore, we evaluated whether the genes harboring the damaging DNVs in schizophrenic patients enrolled in the current study were nominated by recent exome sequencing studies in schizophrenia^8^,^11^, and whether any of identified genes corresponded with the location of the 108 independent schizophrenia-associated loci in the most recent large-scale genome-wide association study (GWAS)^2^. Finally, we tested the association between the number of amalgamated DNVs and cognitive measurements (the anti-interference ability and execute functions) in patients with schizophrenia.

## Results

### *De novo* mutations identified by exome sequencing

We completed exome sequencing in 45 Han Chinese schizophrenia-parent trios. Details are showed in Table S1. On an average, we obtained 7.97 Gb of mappable sequence data per individual after exome enrichment, targeting ~48.56 Mb from exons and their flanking regions. Overall, 1.6% of the total genome was covered, which represented a fraction corresponding to the NCBI Consensus Coding Sequences database (CCDS). A median of read depth was obtained as 54X, which was higher than the estimated depth (33X)^22^ required for highly accurate downstream heterozygous variant detection. Additionally, 90.69% of the captured target exons were covered by high-quality genotype calls (10X) to ensure good detection sensitivity^22^. The relatedness, based on the called genotypes, was consistent with the kinship (Figure S1), thereby suggesting a good quality of the sequencing data.

After the QC of the called variants, 66 exon DNVs were initially identified in the 45 case-parent trios. Fifty-seven mutations were validated by subsequent Sanger sequencing, which included 47 DNVs (Table 1) and 10 synonymous variants (Table S2) in 39 case-parent trios. The ratio of non-synonymous variants to synonymous variants (NS:S) was 4.7:1, which was similar to that reported in previous studies^23^,^24^,^25^,^26^. There were no differences in the sequencing coverage between the trios with sporadic or familial cases, or between the patients with or without *de novo* events (Figure S2). The mutation rate of the *de novo* mutation in the captured exome was estimated to be 2.2 × 10^−8^ mutations per base per generation in the total sample. Of the total DNVs, 45 DNVs were missense variants, and 2 DNVs constituted a single base pair Indel. This Indel led to a coding frame shift at one of the isoforms of the *TRMT112* and *TMEM132A* genes. Of the 47 DNVs, 26 and 21 DNVs occurred in sporadic and familial trios, respectively. The average point mutation rates were 1.7 × 10^−8^ and 3.3 × 10^−8^ mutations per base per generation in 32 sporadic and 13 familial trios respectively. The distribution of DNVs in the 32 sporadic trios followed a Poisson distribution (*p* < 0.14; goodness of fit by likelihood ratio test, mean = 0.81 with 95% confidence interval (CI) [0.5, 1.1]) (Figure S3). The DNVs in the 13 familial trios did not exhibit a Poisson distribution, which was probably attributed to inadequate sample size.

### Prediction for pathogenic potential of DNVs

All the 47 validated DNVs were either absent or had a MAF (<1%) in the reference databases (1000 Genomes Project, dbSNPv137, and NHLBI GO ESP6500). Forty DNVs in 40 different genes were predicted to be damaging, deleterious, or disease causing by at least one of the seven bioinformatics prediction algorithms (SIFT, Polyphen2_HDIV, Polyphen2_HVAR, LRT, MutationTaster, MutationAssessor, and FATHMM) (Table 1). To simplify the annotation, the 40 DNVs were called as damaging mutation throughout the paper unless otherwise specified, and genes with DNV were called as case genes.

### Prediction for pathogenic potential of genes

We then investigated whether the 40 genes harboring the damaging DNVs exhibited pathogenic potentials according to several recent gene-level measurements, along with analyzing co-expression network of these genes in different brain regions at different developmental stages. Evidences from the recent largest GWAS^2^ and previous exome sequencing studies^8^,^11^ were also explored.

### Haploinsufficieny, Recessive Probability and Residual Variation Intolerance Scores (RVIS) analysis

In this study, the average haploinsufficiency score of the 40 genes was 0.37, which was significantly larger than a random set of genes with the same size on the whole genome (empirical *p* = 0.04 using random sampling without replacement). This significance suggests the 40 genes have larger pathogenic potential to cause a dominant disease^27^. Their average recessive probability was not significantly different from a random set of genes (empirical *p* = 0.07 using random sampling without replacement), which suggests many of these genes are unlikely to cause a recessive disease. This pattern is consistent with our hypothetic inheritance model (dominant model)^28^. Their average RVIS (−0.75 and −0.6, which were derived from 0.1% or 1% variants in the ESP6500 sample respectively) were significantly smaller than the random set of the same size (*p* < 3.4 × 10^−6^ and 1.7 × 10^−4^, respectively, using the same random sampling approach)^29^ (Table S3), suggesting that these genes are less tolerant to mutations and are more likely to be pathogenic.

### Brain-critical-exon gene enrichment analysis

Among the 40 genes harboring the damaging DNVs, eight genes (*ABCA2*, *CDC42BPB*, *USP48*, *MACF1*, *CEP170*, *NAV2*, *RNFT2*, and *DICER1*) were enriched for ‘Brain-critical exons’, which are highly expressed in human brain under strict purifying selection^30^. According to hypergeometric distribution test, these eight genes were overrepresented (p < 0.026) in a list of 1744 genes enriched with ‘brain-critical exons’ by Uddin *et al*.^30^. The *ABCA2* gene exhibited the highest exon score, 5.73. The significant enrichment within ‘Brain-Critical Exons’ also implicates the pathogenic potential of these genes to schizophrenia.

### Co-expression of genes harboring *de novo* mutations in different brain regions at different developmental stages

**Figure 1:**
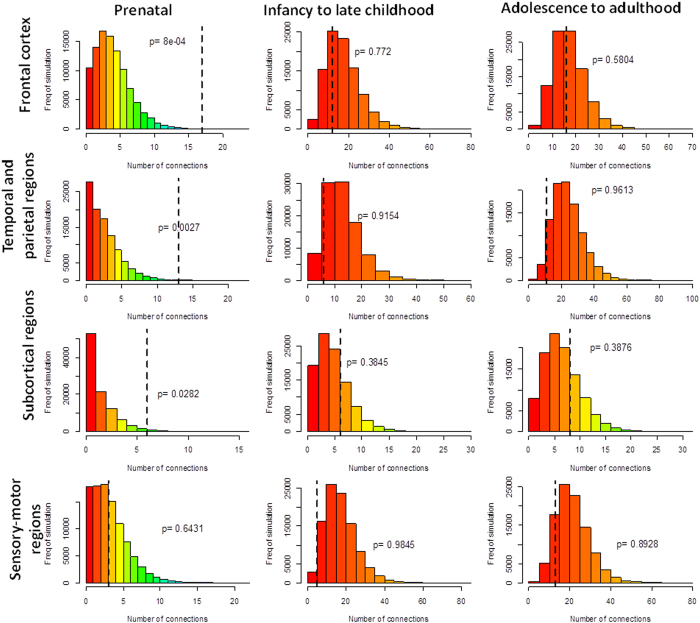
Relatedness of 40 genes in the co-expression network in four brain regions at three developmental stages. We evaluated the co-expression of genes harboring the damaging DNVs in cases and controls using RNA-seq data from the BrainSpan Atlas. Gene pairs were defined as co-expressed if Spearman’s rank correlation coefficient (|R|)> 0.8 for RNA-seq expression levels across different brain areas and a given developmental stage. Networks were created for co-expressed gene pairs as described for Fig. 2. Histograms represent distributions of the numbers of edges in 100,000 simulated networks using genes harboring the damaging DNVs in controls. Dotted lines indicate the numbers of connections (edges) in networks created using genes harboring the damaging DNVs in cases with schizophrenia. The significant enrichment in co-expression of gene mutants in schizophrenia was observed in the frontal cortex and temporal and parietal regions during prenatal development (*p* < 0.0091 and 0.03 after Bonferroni corrections for multiple comparisons) at prenatal stage. There was no enrichment of co-expression of genes harboring the damaging DNVs at the other stages.

Inspired by Gulsuner *et al*.^11^ we subsequently determined whether the 40 genes exhibited significant co-expression in four brain regions (frontal cortex, temporal and parietal regions, sensory-motor regions, and subcortical regions) at three developmental stages (prenatal, infancy to late childhood, and adolescence to adulthood) using RNA-Seq data in the BrainSpan Atlas (Table S4). In the prenatal frontal cortex, the 40 genes harbouring the predicted damaging DNVs in patients showed substantially higher degree of co-expression (*r* ≥ 0.8) as compared to random gene set, matched for gene size from 240 genes harbouring the DNVs (nominal *p* < 8.0 × 10^−4^; Bonferroni correction for multiple comparisons, *p* < 9.1 × 10^−3^; Fig. 1). This finding was consistent with the results reported by Gulsuner *et al*.^11^, although none of the genes harbouring the DNVs overlapped between these two studies. In addition, we also identified a new significant relatedness in the genes that harbour the damaging DNVs (case genes), in the prenatal temporal and parietal regions (nominal *p* < 2.7 × 10^−3^; Bonferroni correction for multiple comparisons, *p* < 0.03), which was not significant in the study of Gulsuner *et al*.^11^ Consistent with Gulsuner *et al*. no other regions or developmental stages exhibited significant relatedness in the co-expression networks. Moreover, co-expression among the five genes harbouring *de novo* benign mutations in the schizophrenia trios was not observed.

**Figure 2:**
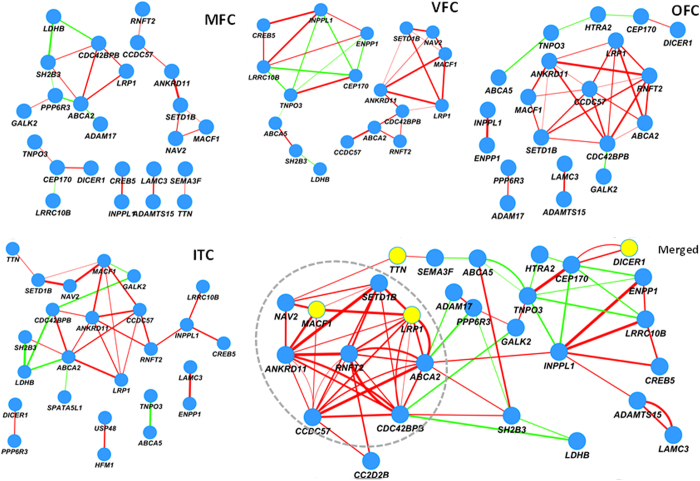
Co-expression networks of four significant subregions in prenatal brain. Each node indicates a gene and each edge denotes a co-expression between a pair of genes. A red edge denotes a positive co-expression, whereas the green edge denotes a negative co-expression. Genes marked in yellow are reported to harbor the damaging DNVs in schizophrenia patients in previous studies. The dashed gray ellipse labels a clique in which the genes are highly connected. ITC: Inferolateral temporal cortex (area TEv, area 20); Merged: Merged network of the four networks; OFC: Orbital frontal cortex; MFC: Anterior (rostral) cingulate (medial prefrontal) cortex; and VFC: Ventrolateral prefrontal cortex.

In the subsequent analysis, we further examined the enrichment for relatedness of the 40 genes in co-expression networks at seven specific anatomical subregions of the prenatal frontal cortex and temporal-parietal regions, including the dorsolateral prefrontal cortex (DFC), anterior (rostral) cingulate (medial prefrontal) cortex (MFC), orbital frontal cortex (OFC), ventrolateral prefrontal cortex (VFC), inferolateral temporal cortex (area TEv, area 20) (ITC), posterior (caudal) superior temporal cortex (area TAc) (STC), and posteroinferior (ventral) parietal cortex (IPC). In accordance with Gulsuner *et al*.^11^, the VFC exhibited significantly greater relatedness in the co-expression network as compared to the genes which do not harbour the damaging DNVs (control genes) (nominal *p* < 4.0 × 10^−3^; Bonferroni correction for multiple comparisons, *p* < 0.048; Table 2 and Fig. 1). Moreover, three subregions (MFC, OFC, and ITC) exhibited significant enrichment of the relatedness in the co-expression networks, i.e., the corrected *p* for multiple comparisons by Bonferroni method was < 0.05. Interestingly, after merging the four networks, all the co-expression pairs were connected in a single network, which contained 28 genes (Fig. 2). In the merged network, nine genes (*NAV2, CDC42BPB, ANKRD11, SETD1B, MACF1, ANKRD11, RNFT2, LRP1, and ABCA2*) were highly inter-connected with positive co-expression (r ≥ 0.8; Fig. 2). In addition, six genes (*ABCA2*, *ANKRD11*, *ANKRD11*, *CDC42BPB*, *RNFT2*, and *LRP1*) exhibited nine or more connections with other genes (Fig. 2).

Furthermore, we demonstrated that of the total 40 genes, excluding two genes lacking expression data in the frontal and temporal cortices, most of the genes exhibited high expression levels in brain in early foetal development, decreased expression at the end of foetal development and during childhood, and an increase in expression during early adulthood. The expression levels of individual network genes in the foetal frontal and temporal cortices are provided in Figures S4 and S5.

### Constrained gene analysis

A statistical measurement, constrained gene score, was recently proposed to evaluate excesses of *de novo* mutations in a gene for human diseases^31^. Subsequently, we asked whether the genes with damaging *de novo* mutations identified in present study are significantly enriched in a list of constrained genes from Kaitlin *et al*.^31^. Among the 40 genes, 8 genes (*LRP1*, *ABCA2*, *CEP170*, *HTRA2*, *DICER1*, *MACF1*, *ADAMTS15* and *SEMA3F*) occurred in the top 1000 constrained gene list (*p* = 9.8 × 10^−4^, hypergeometric test based on 18,988 CCDS protein coding genes) (Table S3), suggesting it is unlikely that these genes harbor *de novo* mutations only by chance in our schizophrenic patients, and the occurrence of *de novo* mutations may be related to schizophrenia. Interestingly, among 8 constrained genes, the top gene *LRP1* has significant positive co-expression with the second gene *ABCA2* in MFC, OFC and ITC. The *LRP1* also has significant positive co-expression with a constrained gene *MACF1* in VFC and ITC, and in merged co-expression network. In addition, the third constrained gene *CEP170* has significance positive co-expression with *DICER1* in MFC and OFC (Fig. 2). Finally, the *DICER1* exhibited a significant negative co-expression with *HTRA2* in OFC.

### Genes in previous studies on *de novo* mutations of schizophrenia

We also assessed which of the 40 genes harboured DNVs in the schizophrenic patients were included in five recent exome sequencing studies using a hypergeometric test based on 19,043 known protein coding genes and 733 involved genes. There were four overlapping genes, including *LRP1*, *MACF1*, *DICER1*, and *TTN* (a marginally significant over-representation, *p* < 0.067). *LRP1* was reported to harbour a stop-gain DNV at c.C6600A^16^. *MACF1* was reported to harbour a DNV at c.C12097T^14^; and the DNV detected in the present study was located at c.C6289T of the *MACF1* transcript NM_012090. *DICER1* was reported to harbour a damaging DNV at c.A1126G^32^, and this gene also contained a damaging DNV at c.C1153T. Given its large length in the exon region (>100 kb), *TTN* may not be an interesting candidate as compared to the other three genes. Nevertheless, this gene was reported to harbour DNVs at c.C7061T^32^ and c.A8134G according to the present study. Interestingly, in the above brain subregions of VFC and ITC, merged co-expression network (Fig. 2), both *LRP1* and *MACF1* demonstrated a high co-expression (*r* > 0.89).

### Overlapped genes with large-scale GWAS

We evaluated whether the genes harbouring the damaging *de novo* mutations also corresponded with the location of any of the 108 independent schizophrenia-associated loci in the most recent large-scale GWAS^2^. The *LRP1* gene was located at the 20^th^ genetic loci (chr12:57428314-57682971), and was the only gene of the total 11 candidate genes in this ~250 kb region. Further studies are required to validate its pathogenic contribution to schizophrenia.

### Integrated evidences for pathogenic prediction of genes with DNVs

Overall, we ranked 40 genes according to the numbers of supporting evidences as described above and 8 genes showed more than two supporting evidences (Table S5). On the top of the list, both *LRP1* and *MACF1* genes are supported by 5 out of 6 independent evidences, suggesting their high pathogenic potential for schizophrenia. Both genes are more likely to be pathogenic as they are less tolerant to mutations (RVIS), are in excess of *de novo* mutations (constrained gene score), and are hub genes in brain specific co-expression network that are highly connected with other genes. Both genes are also reported in previous *de novo* mutation studies in schizophrenia^9^,^11^. In addition, the *MACF1* gene is enriched significantly within brain specific critical exons, and the *LRP1* gene ranked top 20 in list of schizophrenia-associated loci in the recent large-scale GWAS^2^.

### Pathway analysis of genes carrying *de novo* damaging mutations

In Gene ontology (GO) annotation, we observed that eight GO terms (GO:0005524, GO:0000166, GO:0007163, GO:0006796, GO:0004386, GO:0016887, GO:0005911, and GO:0051015) were significantly overrepresented with some of the 40 genes with damaging DNVs (Bonferroni corrected p ≤ 0.05, hypergeometric distribution test; Figure S6). Moreover, physical interaction between *MACF1* and *DISC1* was detected using geneMANIA (http://www.genemania.org/) analysis, where both genes were associated with microtubule-based processes (p < 9.04 × 10–5). Additional details are provided in Figure S6.

### Relationship between the amalgamated DNVs and cognitive function

We also examined the relationship between the amalgamated DNVs and cognitive measurements in schizophrenic patients using a linear regression model. Interestingly, although the association test was generally underpowered due to the small sample, three cognitive measurements [completion time obtained from the Stroop colour test and Stroop colour-word test and perseverative errors obtained from the Modified Wisconsin Card Sorting Test (WCST-M)], which assessed the anti-interference ability and executive functions of the patients with schizophrenia, showed promising associations with *p* values of 0.0455, 0.00995 and 0.0301, respectively. The patients with more DNVs showed worse performance in anti-interference ability and executive function and details were showed in Figure S7; however, these associations must be validated in larger independent samples.

## Discussion

**Figure 3:**
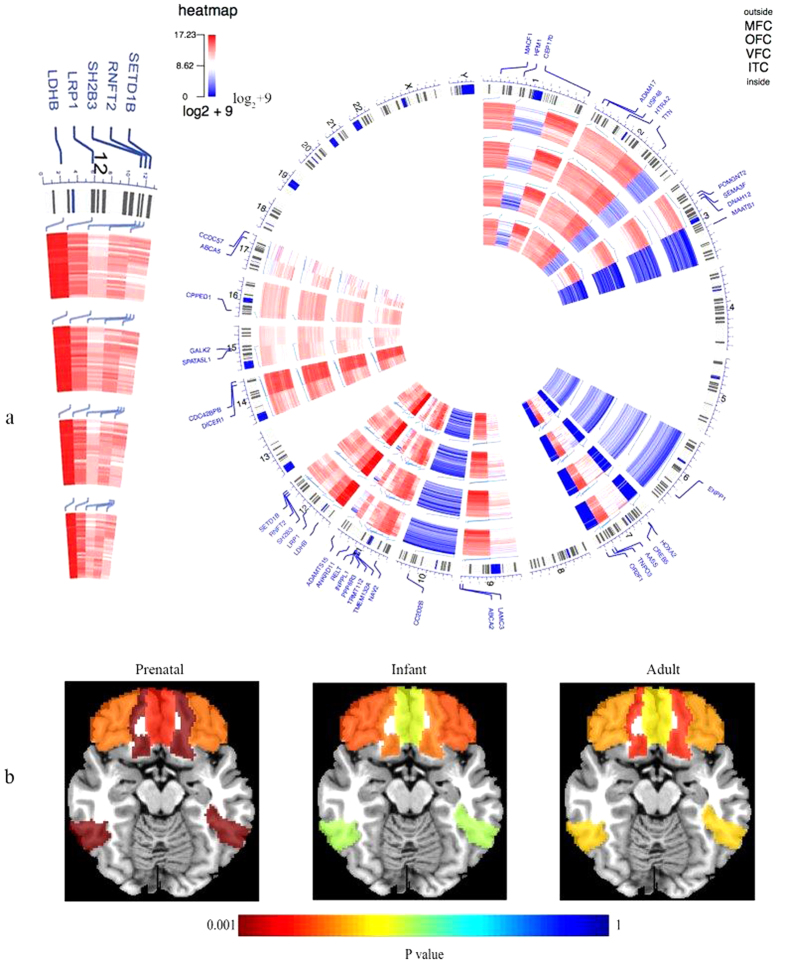
Abstract plot of main findings of the study. (**a**) Circos imaging showing human chromosome ideogram with data tracks for gene labels and their expressional level in different developmental stages, which were transformed with log2(value) + 9 in four brain regions (MFC,OFC,VFC, and ITC), (**b**) Heatmap showing the above four brain regions in three developmental stages and their corresponding p values in co-expression network analyses.

To our knowledge, this is among the first exome sequencing study of schizophrenia in Han Chinese population. Firstly, by analyzing the genomic features, we found that 40 genes harboring the predicted damaging DNVs exhibited pathogenic potentials, which were supported by multiple gene-level assessments, and some of genes were also identified by the recent largest GWAS^2^ and previous exome sequencing studies^9^,^11^. Secondly, we found that the 40 genes harbouring the damaging DNVs in patients showed substantially higher degree of co-expression the prenatal frontal cortex, and in the prenatal temporal and parietal regions. Most of the genes exhibited high expression levels in brain in early foetal development, decreased expression at the end of foetal development and during childhood, and an increase in expression during early adulthood. Finally, we reported that the patients with more amalgamated DNVs showed worse performance in anti-interference ability and executive function in a preliminary analysis. The main findings are summarized in Fig. 3.

Of 8 genes showing more than two supporting evidences for pathogenic prediction, some of genes (e.g. *LRP1*, *MACF1*, *DICER1* and *ABCA2*) harbours *de novo* mutations provided promising evidence as a strong candidate genes for schizophrenia. The *ABCA2* is a hub gene in the co-expression network of prenatal frontal cortex. Importantly, three of these genes (*LRP1*, *MACF1*, and *DICER1*) were found to overlap with the genes associated with schizophrenia in previous studies^10^,^33^,^34^,^35^. *LRP1* binds to neuroserpin and is involved in both serpin internalization and signal transduction. Moreover, neuroserpin expression is up-regulated in a novel *in vitro* model of schizophrenia^36^. Study on induced pluripotent stem cell neuron, derived from patients with schizophrenia, has demonstrated a 5.6-fold increase in the neuroserpin expression as compared to matched controls, while decreased neuronal connectivity was associated with reduced dendritic arborization and impaired synaptic maturation as reported *in vivo*^37^,^38^.

In this study, we found that the genes with damaging DNVs enrich in co-expression networks of prenatal frontal and temporal cortex. Our findings also demonstrated that, in determining the risk of genes for schizophrenia, not only the tissue where the mutated genes express, but also the time when genes expressed may be of critical importance. This result confirms the findings by Xu *et al*.^8^ and Gulsuner *et al*.^11^, and supports a spatial and temporal pattern of DNVs in schizophrenia in ancestrally different samples with different set of genes. Xu *et al*. reported that the damaging DNVs in probands with schizophrenia enriched in a network of genes co-expressed in fetal hippocampus and dorsolateral prefrontal cortex^14^. Gulsuner *et al*. demonstrated damaging DNVs in probands with schizophrenia converged in a network of genes co-expressed in the dorsolateraland ventrolateral prefrontal cortex during fetal development^11^. In addition, we provided preliminary evidence that the genes harboring the DNVs might participate in worsening of neurocognitive performances, such as anti-interference and executive functions. All of these finding supported the hypothesis that disruptions of some vital brain regions at prenatal stage during neurogenesis are critical to the pathophysiological mechanisms of schizophrenia, and the disruption of gene function in some of the brain regions in prenatal stage might contribute to the development of this neurodevelopmental disorder.

Previous studies have showed that anatomical and functional abnormity of prefrontal^39^,^40^, temporal cortex^41^,^42^,^43^, and frontal-subcortical circuit^32^,^44^,^45^,^46^ are involved in the pathogenesis of schizophrenia. Orbitofrontal circuit lesions characterized by disinhibition lead to personality changes, and anterior cingulate circuit lesions are associated with apathy of schizophrenia^47^. It is well known that prefrontal cortical network managed inputs from the other cortical and subcortical brain regions and have an important role in planning and directing motor activities, affection, neurocognition, and social behavior^48^. Ford *et al*. suggested that reduced fronto-temporal functional connectivity may contribute to the misattribution of the inner thoughts to external voices in schizophrenia^49^. In addition, besides the mesolimbic DA system, cortical DA system was found to play an important role in schizophrenia^45^. The growing evidence has showed that the neurodevelopment deficiencies in amygdala-prefrontal cortical circuit may lead to the dysregulation of DAergic modulation of emotional processing and learning, which contributes to the pathogenic mechanisms of schizophrenia^32^. Furthermore, a variety of cellular pathological findings in frontal-cortical tissue were observed during the postmortem of brain in schizophrenia patients^50^. In keeping with previous studies, our study supports that the fronto-temporal-subcortical circuit dysfunction is associated with the development of schizophrenia, which may be partly due to the presence of DNVs in genes as detected in this study. Four out of the nine genes (*NAV2*, *CDC42BPB*, *MACF1*, and *LRP1*) involved in co-expression network have considerable evidence for their pathogenic roles in the development of schizophrenia^33^,^34^,^51^,^52^. *NAV2* (Neuronal Navigators 2) gene encodes a member of the neuron navigator gene family, and may play a role in cellular growth and migration. In previous studies, the expression of *NAV2* gene was found to be associated with actin cytoskeleton remodeling^53^ and was down-regulated in schizophrenia^54^.

GO terms, which were largely related to the biological function and process especially during the early developmental stages, were significantly enriched with some of the 40 genes with damaging DNVs in pathway analysis of the present study. GO:0051015 bears some resemblance with the category (GO:0051017) found in a previous DNVs study^14^. Both pathways act on the actin filament, which is highly enriched in dendritic spine^55^ and preserve cytoskeleton wholeness during cell development and migration^56^.

Some limitations, such as small sample size and lack of normal controls, should be also addressed in this study. We exploited existing data in the public domains to overcome the potential small-sample-size limitation of the current study. Making use of public control data, we demonstrated a significant co-expression pattern of our damaging DNVs genes in prenatal brains. This is highly consistent with two recent studies^1^,^12^ although few of involved genes are identical. Moreover, a number of genes in present study are also supported significantly by genomic features (for example genes’ mutation constraint^37^, RVIS^36^ and critical exon enrichment^38^). While a larger sample is preferable for schizophrenia study, we believe that the current sample revealed a few important susceptibility genes of schizophrenia. This cost-effective strategy should be encouraged so that more redisposing variants can be identified for schizophrenia.

Taken together, our findings in Chinese schizophrenic patients reinforced the pathogenic role of DNVs, particularly in prefrontal, temporal cortex, and frontal-subcortical circuit during the early developmental stages of schizophrenia. Genetic and clinical heterogeneity provide a challenge for population-based association approaches and may require a more precise definition of phenotypes in the mapping of risk genes, especially regarding the improvements in brain function.

## Material and Methods

### Participants

Details of the participants and clinical assessment procedure are shown in supplementary document and in Table S6. Written informed consent was obtained from all the participants and legal guardian. The study was approved by the ethical committee of West China Hospital of Sichuan University, China. And the methods were carried out in accordance with the approved guidelines.

### Exome sequencing

Genomic deoxyribonucleic acid (DNA) was purified from peripheral blood leucocytes. Deoxyribonucleic acid sample was prepared according to the Illumina Paired-End Sample Preparation Guide (http://supportres.illumina.com/documents/myillumina/e5af4eb5-6742-40c8-bcb1-d8b350bcb964/paired-end_sampleprep_guide_1005063_e.pdf). Targeted enrichment was performed using TruSeq Exome Enrichment Kit (Illumina, San Diego, CA, USA), optimized for Illumina sequencing. Exon-enriched DNA libraries from 135 subjects were individually sequenced on an IlluminaHiSeq2000 at Axeq Technologies (http://www.axeq.com/), which produced 101 base paired-end reads, in accordance with the manufacturer’s instructions.

### Data processing and variant calling

The pipeline for exome data analysis is described in Figure S8. Briefly, sequencing reads were initially mapped to University of California, Santa Cruz (UCSC) human reference genome (hg19) using Burrows-Wheeler Alignment tool (BWA)^57^. Duplicate reads were flagged and removed by Picard-tools (http://picard.sourceforge.net/). The Genome Analysis Toolkit (GATK, version 2.3)^58^ was then used for realignment of the reads around the insertion/deletion (Indel) sites and base quality recalibration. Finally, the GATK was used to detect single nucleotide variants (SNVs) and short Indels from all the trios simultaneously. Variants’ quality assessment (QA) and quality control (QC) were performed on raw and clean variant set repeatedly to make sure the calling process was well performed, otherwise the whole calling process was subjected to revision. Concrete criteria in QA include total number of variants, dbSNP coverage, and transition/transversion (Ti/Tv) ratio. The variants’ quality score recalibration (VQSR) was used for QC. Meanwhile, these variants’ sets were also used to examine sample relatedness in PLINK for quality assessment^59^. KGGSeq (http://statgenpro.psychiatry.hku.hk/kggseq/) was used to extract *de novo* SNVs and Indels with standard QC based on the levels of genotype and variants. Genotypes with Phred-scaled quality score of <30, depth <10X, ≥5% alternative allele supporting reference homozygous genotypes, ≤25% and 70% alternative allele supporting heterozygous and alternative homozygous genotypes respectively were eliminated. Variants with Phred-scaled sequencing quality of <50, mapping quality <20, strand bias >60, call rate <60% and Hardy-Weinberg equilibrium test p < 0.00001 were also excluded. A DNV was called as a variant, which was present in the proband, but absent in either of the parents. The DNVs identified using exome sequencing were validated using standard Sanger sequencing on ABI 3730xl DNA Analyzer by designing custom primers (Sigma) based on ~500 bp of genomic sequence, flanking each variant.

### Annotation of *de novo* variants

KGGSeq^60^ was also used to map the extracted DNVs systematically. The variants were mapped into genes according to three gene definitions (RefGene, KnownGene, and GEncode). Alternative allele frequencies from 1000 Genome Project^61^ and NHLBI Grand Opportunity Exome Sequencing Project were used to annotate the *de novo* variants. A variant was regarded as a non-synonymous mutation as long as it was defined by one of the gene definitions. The non-synonymous variant was annotated for its protein damming or deleteriousness potential by seven *in silico* prediction algorithms (SIFT, Polyphen2_HDIV, Polyphen2_HVAR, LRT, MutationTaster, MutationAssessor, and FATHMM) originally collected in dbNSFP2.4^62^. Papers from National Center for Biotechnology Information (NCBI) PubMed database (http://www.ncbi.nlm.nih.gov/pubmed/), which mentioned a gene harboring interested DNVs and schizophrenia in the title or abstract, were also retrieved automatically by KGGSeq.

### Evaluation of statistical significance for a set of genes

We wrote a java program to assess the statistical significance of a measurement for a set of *m* case genes, harboring DNVs, and compared them to a set of *n* control genes (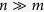). Based on an interested measurement (e.g., pathogenic scores and network connections) as an absolved value, a statistical value, such as mean or summation was first calculated for the case genes. In the control gene set, *t* genes sets with size *m* were randomly sampled without replacement using a random engine in a java package, Colt (http://acs.lbl.gov/ACSSoftware/colt). The same statistical value was calculated for the *t* random gene sets. The number of sets with a statistical value of over or equal to the observed value was represented as *s*. The empirical *p* value was estimated as (*s + 1*)/(*t* + *1*).

### Pathogenic analysis of genes

Three *in silico* scores (haploinsufficiency^27^, recessive probability^28^, and genic intolerance^29^) were employed to accesses the pathogenic impact of genes. The haploinsufficiency and recessive probability scores were downloaded from dbNSFP V2.4^62^. The genic intolerance scores (based on all ESP6500 samples) were downloaded from http://chgv.org/GenicIntolerance/. The significance of these scores for a set of genes harboring interesting DNVs were evaluated by the random sampling approaches as mentioned above.

### Brain-critical-exon gene enrichment analysis

The 1744 genes enriched with ‘brain-critical exons’ were downloaded from the supplementary Table 7 of Uddin *et al*.^30^ to assess the contribution of genes harboring the damaging DNVs. Hypergeometric distribution was used to evaluate the statistical significance of enrichment using our R package (http://www.r-project.org/). In addition, the critical exon-scores of genes in this table were used to prioritize genes with damaging DNVs.

### Constrained genes analysis

We downloaded the top 1003 constrained genes, which were specifically provided to evaluate excesses of *de novo* mutations in a gene for human diseases. In the NHLBI Grand Opportunity Exome Sequencing Project (ESP) sample, which contained about 6500 individuals, these genes tend to have less rare missense variants than the expected, suggesting strong purifying selection in human population. We checked whether the top constrained genes were enriched in our genes harboring *de novo* mutations by the hypergeometric distribution test.

### Gene ontology (GO) annotation

To characterize the functions of genes harboring the damaging DNVs, the gene set enrichment analysis (GSEA) on GO was performed under a hypergeometric distribution on R package (http://www.r-project.org/). The GO surviving Bonferroni correction (*p* < 0.05) was regarded as significant. The GO gene sets were obtained from NCBI (ftp://ftp.ncbi.nlm.nih.gov/gene/DATA/gene2go.gz) and the pathway gene sets from Pathway Interaction Database (PID) (http://pid.nci.nih.gov/).

### Co-expression network analysis

The standardized gene expression data in 26 brain subregions at multiple developmental stages were downloaded from BrainSpan (http://www.brainspan.org). Four regions (frontal cortex, temporal and parietal regions, sensory-motor regions, and subcortical regions), which include 11 subregions, were selected for co-expression analysis at three developmental stages: (1) prenatal stage: 8–37 post conception weeks (PCW); (2) infancy to late childhood: 4 months to11 years; and (3) adolescence to adulthood: 13–23 years according to Gulsuner *et al*.^11^ (Table S4).

A co-expression network was built for each of the brain regions at different developmental stages. Given the expression values for a pair of genes, Spearman’s rank correlation coefficient (*r*) was calculated to measure the level of co-expression of paired genes. Similar to Gulsuner *et al*.^11^ we used a cutoff point (*r* = 0.8) to exclude the genes with relatively low co-expression levels. The retained co-expression gene pairs were used to form a co-expression network. The above random sampling approach was used to evaluate the statistical significance of interconnectedness for non-interested case gene set by counting the connections, or edges^36^, as compared to 240 control genes harboring the damaging DNVs in healthy subjects^11^. Cytoscape 3.0.1^63^ was used to visualize and plot the network.

### Statistical analysis

The Kolmogorov-Smirnov test (KS-test) was used to compare the distribution of prediction scores among DNVs. Fisher’s exact test or Chi-square test with Yates’ correction was used for the analysis of contingency tables depending on the sample sizes. We examined the relationship between the number of DNVs and cognitive measurements using a linear regression model. All statistical analysis was conducted using R^64^.

## Additional Information

**How to cite this article**: Wang, Q. *et al*. Increased co-expression of genes harboring the damaging *de novo* mutations in Chinese schizophrenic patients during prenatal development. *Sci. Rep*. **5**, 18209; doi: 10.1038/srep18209 (2015).

## Supplementary Material

Supplementary Information

## Figures and Tables

**Table 1:** The predicted functions of the 47 validated *de novo* non-synonymous variants used 7 bioinformatics tool.

Chromosome	Position	R/A	Gene	MaxAF	GeneFuture	SIFT	Polyphen2_HDIV	Polyphen2_HVAR	LRT	MutationTaster	MutationAssessor	FATHMM
1	22021668	A/G	*USP48*	.	missense	T	B;B;B;B	B;B;B;B	N	D	N	T;T
1	39827053	C/T	*MACF1*	.	missense	D	B;B;B;B	B;B;B;B	N	D	L	T;T;T;T;T;T
1	91844691	C/T	*HFM1*	.	missense	D	P;D;D	P;D;P	U	D	M	T;T
1	243319624	G/A	*CEP170*	.	missense	.	.	.	.	D	.	.
2	9650202	G/A	*ADAM17*	5.00E-04	missense	T	D;D	D;D	D	D	M	D
2	74758135	T/G	*HTRA2*	.	missense	D	D;D;D;D	D;D;D;D	D	D	M	D;D
2	179635385	T/C	*TTN*	.	missense	D	D;D;D;D;D	D;D;D;D;D	.	D	L	T;T;T;T;T
3	43121633	C/T	*GTDC2*	5.00E-04	missense	T	D	D	D	D	M	D;D
3	50211303	G/C	*SEMA3F*	.	missense	D	P;P	B;B	D	D	L	T;T;T;T;T;T
3	57335855	C/A	*DNAH12*	.	missense	.	.	.	.	N	H	.
3	119462963	C/T	*MAATS1*	3.83E-04	missense	D	D;D;D	D;D;D	D	D	M	T
6	132207739	C/G	*ENPP1*	.	missense	D	D	D	D	D	M	D
7	27140937	A/G	*HOXA2*	.	missense	D	D	D	D	D	H	D
7	28547270	G/T	*CREB5*	.	missense	D	D	D	D	D	M	T;T;T;T;T
7	121773654	C/T	*AASS*	.	missense	T	P	P	D	D	N	T;T
7	128658136	T/G	*TNPO3*	.	missense	D	D;D;D	D;D;D	D	D	M	T;T;T;T
7	143657109	G/A	*OR2F1*	.	missense	D	D	D	D	N	H	T
9	133952647	G/A	*LAMC3*	.	missense	T	D	P	N	N	L	T
9	139907507	G/A	*ABCA2*	1.23E-04	missense	T	B;B	B;B	U	N	N	D;D;D
9	140147394	C/T	*C9orf173*	.	missense	T	B;P;P;B	B;B;B;B	N	N	N	T;T
10	97763961	A/C	*CC2D2B*	.	missense	T	.	.	.	D	.	T
11	20119268	C/T	*NAV2*	.	missense	.	P;P;P;D	P;B;P;B	D	D	N	T;T;T;T;T;T;T;T
11	61277216	C/T	*LRRC10B*	.	missense	T	B	B	U	N	N	T
11	68370926	G/C	*PPP6R3*	.	missense	T	D;B;P;D;D;D;D;P	D;B;P;P;P;P;D;P	D	D	L	T;T;T;T;T;T;T;T;T;T;T
11	71943335	G/A	*INPPL1*	.	missense	T	D	D	D	D	M	T;T;T
11	73103282	G/A	*RELT*	.	missense	D	B	B	N	N	M	T;T
11	130319553	T/G	*ADAMTS15*	.	missense	D	P	P	.	D	L	T
12	21795009	G/A	*LDHB*	.	missense	D	P	P	N	D	H	D;D;D
12	57554800	A/G	*LRP1*	.	missense	T	D	D	D	D	M	D
12	111885301	C/T	*SH2B3*	.	missense	D	D;D;D	D;D;D	D	D	M	T;T
12	117187992	G/A	*RNFT2*	.	missense	T	P;P	B;B	D	D	L	T;T
12	122261146	G/C	*SETD1B*	.	missense	T	P	B	N	N	N	D;D
14	95590756	G/A	*DICER1*	.	missense	D	D	P	D	D	L	T;T;T;T;T
14	103465975	C/T	*CDC42BPB*	.	missense	D	P	P	D	D	L	T
15	45694835	G/A	*SPATA5L1*	.	missense	T	D	P	D	D	M	D
15	49584699	T/A	*GALK2*	.	missense	D	D;D	D;D	D	D	M	D;T;D
16	12798895	G/A	*CPPED1*	.	missense	D	D	D	D	D	M	D
16	89350182	T/C	*ANKRD11*	.	missense	T	B;B	B;B	N	D	L	T;T
17	67302892	T/C	*ABCA5*	.	missense	.	B;P	B;B	D	D	N	D;D
17	80146156	G/A	*CCDC57*	.	missense	T	D;D	D;D	D	D	M	T;T;T
1	152191073	C/T	*HRNR*	.	missense	T	B	B	.	N	N	T
6	42074959	C/T	*C6orf132*	.	missense	T	.	.	.	N	N	T
6	46660790	C/T	*TDRD6*	.	missense	T	B;B	B;B	N	N	L	T;T
11	60703710	CG/C-	*TMEM132A*	.	frameshift	.	.	.	.	.	.	.
11	64084952	A/+C	*TRMT112*	.	frameshift	.	.	.	.	.	.	.
12	21175888	A/C	*SLCO1B7*	.	missense	T	B;B	B;B	N	N	N	T;T;T
20	3674309	C/T	*SIGLEC1*	0.009304	missense	T	B;B	B;B	N	N	N	T;T

R/A: Reference/Alternative alleles; Missing value;MaxAF: maximal alterative allele frequency; SIFT T: tolerated; SIFT D: damaging; Polyphen2B: benign; Polyphen2 D: probably damaging; Polyphen2P:possibly damaging mutation; LRT D: Deleterious; LRT N: neutral;LRT U:unknown; MutationTasterD disease_causing; MutationTasterN: polymorphism; MutationAssessorH: highfunctional impact; MutationAssessor M: medium functional impact; MutationAssessor L: low functional impact; MutationAssessor N: neutral functional impact; FATHMM D: damaging; FATHMM T: tolerated.

**Table 2:** Analysis of networks of 40 genes with predicted damaging *DNVs* in co-expression networks of four brain regions at three developmental stages.

	Prenatal		Infancy to late childhood		Adolescence to adulthood	
	#Node	#Connection(p-value)	#Node	#Connection(p-value)	#Node	#Connection(p-value)
Medial prefrontal cortex (MFC)	24	23 (0.003)	20	21 (0.817)	26	40 (0.706)
Ventrolateral prefrontal cortex(VFC)	18	30 (0.004)	23	37 (0.303)	31	48 (0.477)
Orbital frontal cortex(OFC)	20	29 (0.001)	21	21 (0.465)	27	44 (0.266)
Inferolateral temporal cortex (ITC)	25	31 (0.001)	15	10 (0.853)	26	47 (0.478)

The p-values were estimated from 100,000 random samples in the controls gene set.
