# Supplementary material for: Increased co-expression of genes harboring the damaging de novo mutations in Chinese schizophrenic patients during prenatal development
Source: Sci Rep. 2015 Dec 15;5:18209. doi: 10.1038/srep18209 (PMC4678883; doi:10.1038/srep18209)
Supplement: Supplementary Information [file srep18209-s1.pdf]

Increased co-expression of genes harboring the damaging de novo mutations in Chinese schizophrenic patients during prenatal development

Qiang Wang[1,2]*, Miaoxin Li[3]*, Zhenxing Yang[2,3], Xun Hu[4], Hei-Man Wu[3], Peiyan Ni[2], Hongyan Ren[1], Wei Deng[1], Mingli Li[1], Xiaohong Ma[2], Wanjun Guo[1], Liansheng Zhao[2], Yingcheng Wang[2], Bo Xiang[1], Wei Lei[1], Pak C Sham[3]** and Tao Li[1,2]**

Supplementary Note

Sample characteristics

Forty-five patients with schizophrenia and their unaffected biological parents were recruited from Sichuan province of China. The patients with schizophrenia included 22 males (49%) and 23 females (51%). All participants were interviewed using the Structured Clinical Interview for the DSM-IV (SCID-P for patients and SCID-NP for parents), which was conducted by 2 trained psychiatrists. All patients met diagnostic DSM-IV criteria for schizophrenia. Family psychiatric history was obtained by interviewing the patients and each participating parents, as well as other first-degree relatives (e.g., sibling and offspring) when possible; these individuals provided detailed information regarding family history during the clinical interview. The average age of the patients with schizophrenia at recruitment was 25.23 years (yrs). The average age at disease onset was 24.34 yrs. The average year of education of the patients was 12.21 yrs. The patients with schizophrenia also underwent further clinical evaluation, which included symptoms on the Positive and Negative Syndrome Scale[1] (PANSS) and global functioning on the Global Assessment of Functioning(GAF)[2]. In addition, the psychiatric history of each patient with schizophrenia was reviewed to exclude individuals with a previous history of a major psychiatric disorder (e.g., psychotic, affective and schizoaffective disorders, head trauma, drug abuse and hereditary neurological disorders). The parents of the patients were screened using a systematic questionnaire to exclude a history of psychiatric illness, including psychosis, anxiety, depression or other axis I mental disorders of the DSM-IV.

Evaluation of neurocognitive functions of patients with schizophrenia

In this study, we used a battery of neuropsychological tests to evaluate each subject during a single session for each participant. Briefly, 14 tests in this battery were selected as they may represent five domains of neurocognitive function as suggested by previous studies[3], including attention, memory and learning, verbal function, visual constructive abilities, and executive function and speed of information processing. there are five subtests (Digit symbol, Arithmetic, Information,

Digit span, and Block design) from Wechsler Adult Intelligence Scale – Revised in China[4]; Immediate and delayed logical memory test, and Immediate and delayed Visual Reproduction test from Wechsler Memory Scale – Revised in China[5]. In addition, Trail Making, Part A and Part B-M[6,7], Verbal fluency test [8], Modified Wisconsin Card Sorting Test-Modified[9] (WCST-M; Nelson, 1976) and Tower of Hanoi[10] are included in the battery. Handedness was assessed by the Annett handedness scale[11] for all participants. All neuropsychological tests we used here have been reported in our previous studies, which analyzed neurocognitive deficits in first-episode schizophrenic patients and their first-degree relatives[12,13]. The details of the complete procedure for each test are reported elsewhere[4,5,9,12,14,15].

Pathogenic scores detections for DNVs

Since only part of DNVs to be pathogenic, we limited our functional analyses to DNVs defined by the following categories: (1) KGGSeq[16] was used to map the extracted DNVs systematically; (2) The variants were mapped into genes according to three gene definitions (RefGene, KnownGene and GEncode); (3) Alternative allele frequencies from 1000 Genome Project[17] and NHLBI Grand Opportunity Exome Sequencing Project (https://esp.gs.washington.edu/drupal/) were used to annotate the *de novo* variants. A variant was regarded as a non-synonymous mutation as long as it was supported by one of gene definitions mentioned above; (4) The non-synonymous variant was annotated for its protein damming or deleteriousness potential by seven *in silico* prediction algorithms (SIFT[18], Polyphen2_HDIV[19], Polyphen2_HVAR[19], LRT[20], MutationTaster[21], FATHMM, and MutationAssessor[22] (http://mutationassessor.org/) originally prepared by dbNSFP2.4[23]). KGGSeq also automatically retrieve published papers from National Center for Biotechnology Information PubMed database (http://www.ncbi.nlm.nih.gov/pubmed/) that mentioned a gene harbouring interested DNVs and schizophrenia in the title or abstract.

Evaluation of Haploinsufficiency Score, Recessive Probability, and Residual Variation Intolerance Scores (RVIS)

We used the gene-based probability of exhibiting haploinsufficiency from supplementary data set 2

generated by Huang et al [24] to evaluate the potential intolerance of genes with DNV in the mutational class groupings. We compared the distribution of haploinsufficiency probabilities for genes in the defined mutational categories to the rest of the genome using a two-sided Wilcoxon rank sum test. We found that genes with *de novo* nonsense mutations ranked significantly higher with greater probabilities of haploinsufficiency. The genes with nonsense DNVs were ranked with high probabilities of haploinsufficiency as showing in Table S3.

We used the method proposed by MacArthur et al. to evaluate the recessive probability of 40 genes harbouring damaging DNVs[25], by evaluating all of the variation in a gene compared to a well-validated null model (recessive model) in order to identify genes with an excess of potentially pathogenic variants and prioritize candidate genes for clinical sequencing studies in clinical disorders.

It has been suggested that genes carrying fewer common functional variants in population may be predicted to cause certain kinds of disease[26]. Petrovski et al. proposed that RVIS, in which the total number of common variants is regressed by the total number of protein-coding variants (including synonymous variants, regardless of frequency in the population) observed within a gene, can be used to evaluate the potential pathogenic risk of genes harbouring DNVs. Subsequently, it can facilitate interpreting personal genomes and identifying high impact mutations through the gene in which they occur[26].

Brain-critical exon scores

Under purifying selection, the genes harbouring deleterious DNVs are difficult to transmit to next generation because of high effect sizes. In order to escape the pressure of selection, high expression exon in brain cannot have high burden of rare missense mutations. So, the specific exons (brain-critical exons), in which there are inverse correlations between their expression level in brain and burden of rare missense mutations, could be deleterious[27]. Uddin et al. found that brain-critical exons under purifying selection should be prioritized in genotype-phenotype studies for autism spectrum disorder and related neurodevelopmental conditions, including schizophrenia. 3955 brain-critical exons in 1744 genes were detected in their study[28] .

Gene ontology (GO) annotation

In gene ontology annotation, we determined that 8 GOs were significantly overrepresented in the 40 genes with predicted damaging DNVs (Bonferroni corrected $p \leq 0.05$, hypergeometric distribution test) (see Figure S7). Of these GO terms, GO:00051015 is related mainly to actin filament binding and functions interactively with other molecule to keep cytoskeleton wholeness during cell development and migration[29]. Moreover, Analysis by geneMANIA (http://www.genemania.org/) detected physical interaction between *MACF1* and *DISC1* with both of these 2 genes being involved in function of microtubule-based process ($P < 9.04 \times 10^{-5}$). Although other terms were not reported to be directly involved in schizophrenia *per se,* some of them are linked to cell growth and DNA synthesis, such as nucleotide binding (GO:0000166), helicase activity (GO:0004386) and ATP-binding (GO:0005524), others like cell-cell junction (GO:0005911) and establishment of polarity linked to cell migration (GO:0007163).

The GO terms significantly enriched with genes harbouring damaging DNVs detected in this study are largely related to biological function and process in the early developmental stage, which is consistent with the fact that disruption of neural growth in schizophrenia happens as early as in the prenatal stage. GO:0051015 bears some resemblance to the category found in a DNVs study (GO:0051017) [30]. Both pathways work on actin filament which is highly enriched in dendritic spine[31] and keep cytoskeleton wholeness during cell development and migration[29]. Moreover, the dendritic spine that is one of the most important cytoskeletal components in the neural circuits of cortex was shown to interact profoundly with actin filament and to be significantly disrupted in schizophrenia patients [31,32]. Dendritic spine has also been shown to be significantly defective in dorsal prefrontal context (DPFLC) of schizophrenic patients[32]. GO:00051015 is related mainly to actin filament binding while GO:00051017 is related to actin filament bundling. Both molecules function interactively to keep cytoskeleton wholeness during cell development and migration[29]. Another evidence worth mentioning is that many by-far-known candidate genes of schizophrenia like *DISC1* and dysbindin take effect based on interfacing with actin cytoskeleton or cytoskeletal proteins such as gird[33]. Furthermore, one other study detected interaction among *MACF1*, *DISC1* and dysbindin, which indicated that possible parts of *MACF1* mutants played on neruodevelopmental pathway of schizophrenia was likely materialized in structure and function of

synapses by disruption of intracellular transportation and cytoskeletal stability[34]. Besides spatial feature, two studies have already identified the temporal relationship of expression profile of enriched GO term with neuron development.

Supplementary Tables

Table S 1: Overview of Exome sequencing data

	Average	Percent
Total reads	78948572	100.00%
Initial mappable reads	78797463	99.81%
Non-redundant unique reads	60449248	76.57%
On-target reads	41650000	52.76%
> 1x coverage	58974193.95	94.99%
>10 x coverage	56304550.4	90.69%
Mean depth of target region	54 x	
Number of SNPs	76382	
Number of coding SNPs	20439	
Number of synonymous SNPs	10608	
Number of nonsynonymous SNPs	9306	
Number of indels	7854	
Number of coding indels	401	

Table S 2: The 10 validate *de novo* synonymous variants

	Position (hg19)	R/A	rsID	Gene	GeneFeature	MaxAF
1	207850750	G/A	.	*CR1L*	synonymous	.
6	16328251	G/A	.	*ATXN1*	synonymous	.
6	33644571	C/T	.	*ITPR3*	synonymous	4.54E-04
7	72891976	C/T	.	*BAZ1B*	synonymous	1.16E-04
7	135333190	A/G	.	*NUP205*	synonymous	.
9	73240202	G/A	.	*TRPM3*	synonymous	.
10	32197661	G/T	.	*ARHGAP12*	synonymous	.
15	51397257	C/T	rs151221769	*TNFAIP8L3*	synonymous	4.55E-04
19	621784	C/T	.	*POLRMT*	synonymous	.
22	17444690	A/G	.	*GAB4*	synonymous	.

Table S 3: Prediction of Pathogenic Score in this study

Gene	Position (Hg19)	R/A	RVIS rank	Haploinsufficiency Scores	Reccessive Scores	Exon Score	Constrained Gene Score
LRP1	57554800	A/G	-7.28 (0.02%)	0.726|11.4%	0.7664|295	-	7.67536| 0.03%
ABCA2	139907507	G/A	-4.24 (0.12%)	0.464|30.9%	0.2982|2042	5.7325	5.8465| 0.11%
CEP170	243319624	G/A	-0.27 (34.82%)	0.444|18.4%	0.1230|6655	0.4410	4.2060| 0.99%
HTRA2	74758135	T/G	0.28 (71.27%)	0.760|9.8%	0.3785|1467	-	3.678807| 2.3%
DICER1	95590756	G/A	-1.52 (3.44%)	0.389|22.0%	0.4949|929	0.4410	3.5382| 2.9%
MACF1	39827053	C/T	-3.92 (0.21%)	0.384|22.3%	-	0.4410	3.53394| 2.9%
ADAMTS15	130319553	T/G	0.68 (84.75%)	0.425|35.2%	0.1457|5604	-	3.531334| 2.9%
SEMA3F	50211303	G/C	-0.26 (34.88%)	0.527|24.8%	0.1486|5421	-	3.140365| 4.9%
USP48	22021668	A/G	-0.56 (19.73%)	0.192|80.2%	0.1021|10806	0.8819	-
HFM1	91844691	C/T	-0.72 (14.27%)	0.144|91.1%	-	-	-
ADAM17	9650202	G/A	0.6 (82.78%)	0.241|36.6%	0.3813|1453	-	-
TTN	179635385	T/C	2.17 (98.04%)	0.352|24.8%	0.3842|1437	-	-
GTDC2	43121633	C/T	-0.88 (10.54%)	-	-	-	-
DNAH12	57335855	C/A	0.84 (88.36%)	0.364|24.0%	0.0998|11196	-	-
MAATS1	119462963	C/T	-	-	-	-	-
ENPP1	132207739	C/G	-0.4 (26.98%)	0.555|22.3%	0.4715|1026	-	-
HOXA2	27140937	A/G	-0.34 (30.07%)	0.468|30.3%	0.1388|6033	-	-
CREB5	28547270	G/T	-0.67 (15.62%)	0.772|6.5%	-	-	-
AASS	121773654	C/T	-0.91 (10.12%)	0.450|18.1%	-	-	-
TNPO3	128658136	T/G	-1.18 (5.94%)	0.645|15.8%	0.1144|8318	-	-
OR2F1	143657109	G/A	0.2 (67.19%)	-	0.0831|13176	-	-
LAMC3	133952647	G/A	0.64 (83.64%)	0.250|65.4%	-	-	-
C9orf173	140147394	C/T	0.57 (82.08%)	-	-	-	-
CC2D2B	97763961	A/C	0.35 (74.37%)	0.362|24.2%	-	-	-
NAV2	20119268	C/T	-1.56 (3.22%)	0.363|24.1%	0.1109|9031	0.4410	-
LRRC10B	61277216	C/T	-	-	-	-	-
PPP6R3	68370926	G/C	0.04 (57.41%)	-	-	-	-
INPPL1	71943335	G/A	-1.63 (2.84%)	0.213|75.1%	-	-	-
RELT	73103282	G/A	0 (54.03%)	0.235|69.5%	0.2474|2658	-	-
LDHB	21795009	G/A	-0.25 (35.75%)	0.468|17.1%	0.8506|178.5	-	-
SH2B3	111885301	C/T	-	0.749|10.2%	0.3582|1595	-	-
RNFT2	117187992	G/A	-0.8 (12.24%)	0.273|59.8%	-	0.4410	-
SETD1B	122261146	G/C	0.2 (67.43%)	0.189|45.1%	0.1062|10039	-	-
CDC42BPB	103465975	C/T	-3.29 (0.42%)	0.338|47.5%	0.1160|8114	0.8819	-
SPATA5L1	45694835	G/A	-0.22 (37.54%)	0.304|53.5%	0.0896|12577	-	-
GALK2	49584699	T/A	-0.18 (40.36%)	0.333|48.5%	0.1410|5888	-	-
CPPED1	12798895	G/A	1.29 (93.84%)	-	-	-	-
ANKRD11	89350182	T/C	-4.38 (0.09%)	0.207|41.9%	0.1074|9788	-	-
ABCA5	67302892	T/C	0.77 (86.96%)	0.255|64.1%	0.1135|8421	-	-
CCDC57	80146156	G/A	-	0.103|71.5%	-	-	-

HRNR	152191073	C/T	-	0.158|51.9%	-	-	-
C6orf132	42074959	C/T	-	0.171|49.0%	-	-	-
TDRD6	46660790	C/T	-0.66 (16.08%)	0.248|66.0%	0.0961|11731	-	-
TMEM132A	60703710	CG/C-	-0.44 (24.71%)	0.296|55.1%	0.0996|11238	-	-
TRMT112	64084952	A/+C	-0.08 (47.79%)	-	-	-	-
SLCO1B7	21175888	A/C	1.71 (96.47%)	-	-	-	-
SIGLEC1	3674309	C/T	-1.81 (2.19%)	0.145|90.8%	0.2083|3372	-	

Table S 4 Number of Tissues from the BrainSpan Atlas Used in Network Analyses

Brain region	Abbreviation	Tissue	Number of specimens		
			8-37 pcw	4 months-11 years	13-23 years
SC	STR	Striatum	14	9	7
	MD	Mediodorsal nucleus of thalamus	8	11	7
	AMY	Amygdaloid complex	14	13	8
	HIP	Hippocampus	15	12	8
SM	A1C	Primary auditory cortex (core)	14	12	8
	M1C	Primary motor cortex (area M1, area 4)	10	11	8
	S1C	Primary somatosensory cortex (areas S1,3,1,2)	10	11	8
	V1C	Primary visual cortex (striate cortex, area V1/17)	15	13	7
FC	DFC	Dorsolateral prefrontal corte	17	13	7
	MFC	Anterior (rostral) cingulate (medial prefrontal) cortex	15	13	8
	OFC	Orbital frontal cortex	14	12	8
	VFC	Ventrolateral prefrontal cortex	16	14	8
TP	ITC	Inferolateral temporal cortex (area TEv, area 20)	13	15	9
	STC	Posterior (caudal) superior temporal	14	16	9

		cortex (area TAc)			
	IPC	Posteroinferior (ventral) parietal cortex	14	13	9

Note: Abb.: Abbreviation. SC: sub-cortical regions, SM: sensory-motor regions, FC: frontal cortex, TP: temporal-parietal cortex. 8-37 pcw (post conception weeks): prenatal stage, 4 months – 11 years: infancy to late childhood,

Table S 5 Summary table of genes supported by multiple evidences

Gene	Constrained Gene (5%)[a]	RVIS (5%)[b]	Hub Gene In Bain Specific Coexpression Network (d≥4)	Bain Specific Critical Exon	In Previous DeNovo Mutation Study	In Previous GWAS	Positive Count
LRP1	+	+	+	-	+	+	5
MACF1	+	+	+	+	+	-	5
DICER1	+	+	-	+	+	-	4
ABCA2	+	+	+	+	-	-	4
CDC42BPB	-	+	+	+	-	-	3
CEP170	+	-	-	+	-	-	2
NAV2	-	+	-	+	-	-	2
RNFT2	-	-	+	+	-	-	2

Note: a, the percentage indicates the top 5% of constrained genes; b, the percentage indicates the top 5% of RVIS.

Table S 6: Clinical and Demographic information of Participants

Family ID	Family type	Race/ ethnicity	Gender	Age	Paternal age	Maternal age	FH *	Paternal age at patient birth	Maternal age at patient birth
Trios1	Trios	Han Chinese	F	29	57	54	No	28	25
Trios2	Trios	Han Chinese	M	18	46	45	No	28	27
Trios3	Trios	Han Chinese	F	23	50	48	No	27	25
Trios4	Trios	Han Chinese	F	20	46	46	No	26	26
Trios5	Trios	Han Chinese	F	26	51	49	No	25	23
Trios6	Trios	Han Chinese	M	21	51	50	No	30	29
Trios7	Trios	Han Chinese	F	18	43	40	No	25	22
Trios8	Trios	Han Chinese	M	23	50	48	No	27	25
Trios9	Trios	Han Chinese	M	16	45	42	No	29	26
Trios10	Trios	Han Chinese	F	20	45	43	No	25	23
Trios11	Trios	Han Chinese	M	16	38	38	No	22	22
Trios12	Trios	Han Chinese	M	19	43	46	No	24	27
Trios13	Trios	Han Chinese	F	20	42	45	No	22	25
Trios14	Trios	Han Chinese	F	19	43	43	No	24	24
Trios15	Trios	Han Chinese	M	28	56	58	No	28	30
Trios16	Trios	Han Chinese	M	18	45	45	No	27	27
Trios17	Trios	Han Chinese	M	18	40	41	No	22	23
Trios18	Trios	Han Chinese	M	17	41	40	No	24	23
Trios19	Trios	Han Chinese	M	23	49	48	No	26	25
Trios20	Trios	Han Chinese	F	21	45	47	No	24	26
Trios21	Trios	Han Chinese	M	16	42	41	No	26	25
Trios22	Trios	Han Chinese	M	23	56	50	No	33	27
Trios23	Trios	Han Chinese	F	19	48	43	No	29	24
Trios24	Trios	Han Chinese	M	22	50	50	No	28	28
Trios25	Trios	Han Chinese	F	18	44	46	No	26	28
Trios26	Trios	Han Chinese	M	23	49	48	No	26	25
Trios27	Trios	Han Chinese	F	23	47	50	No	24	27
Trios28	Trios	Han Chinese	M	19	47	45	No	28	26
Trios29	Trios	Han Chinese	M	21	54	53	No	33	32
Trios30	Trios	Han Chinese	F	16	41	40	No	25	24
Trios31	Trios	Han Chinese	M	22	43	45	No	21	23
Trios32	Trios	Han Chinese	F	17	39	36	No	22	19
Trios33	Trios	Han Chinese	M	16	41	37	Yes	25	21
Trios34	Trios	Han Chinese	F	30	56	57	Yes	26	27
Trios35	Trios	Han Chinese	F	28	64	57	Yes	36	29

Trios36	Trios	Han Chinese	M	21	49	49	Yes	28	28
Trios37	Trios	Han Chinese	F	16	61	36	Yes	45	20
Trios38	Trios	Han Chinese	F	17	40	36	Yes	23	19
Trios39	Trios	Han Chinese	M	33	61	62	Yes	28	29
Trios40	Trios	Han Chinese	M	23	49	50	Yes	26	27
Trios41	Trios	Han Chinese	F	18	41	41	Yes	23	23
Trios42	Trios	Han Chinese	M	17	53	50	Yes	36	33
Trios43	Trios	Han Chinese	M	17	38	38	Yes	21	21
Trios44	Trios	Han Chinese	F	20	41	41	Yes	21	21
Trios45	Trios	Han Chinese	M	23	48	47	Yes	25	24

＊FH: family history

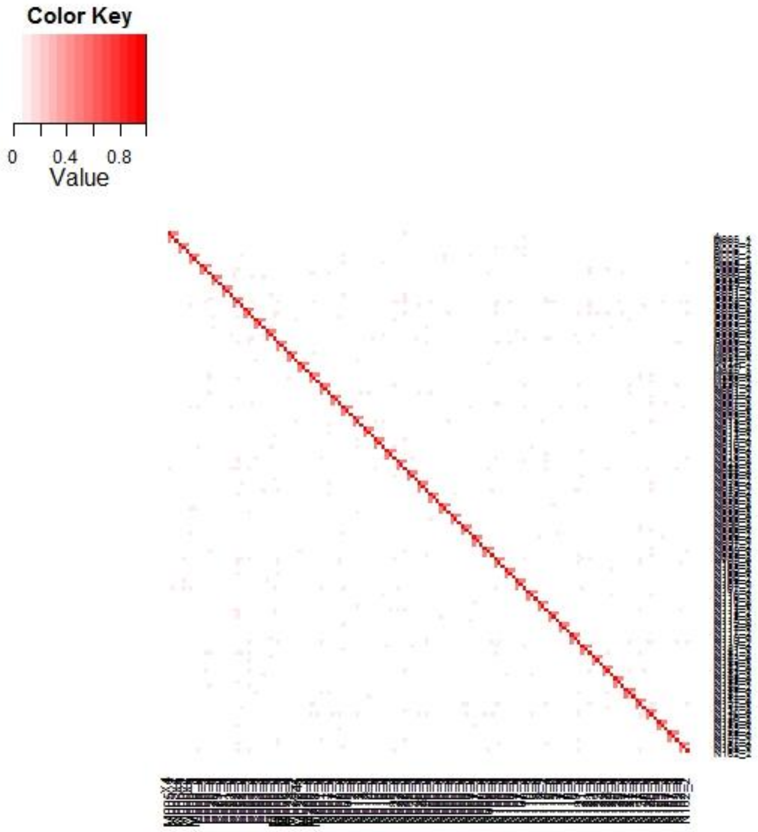

Figure S 1: the relatedness based on the called genotypes between individuals

The figure showed that the relatedness based on the called genotypes was consistent with the kinship, suggesting very good quality of the sequencing data.

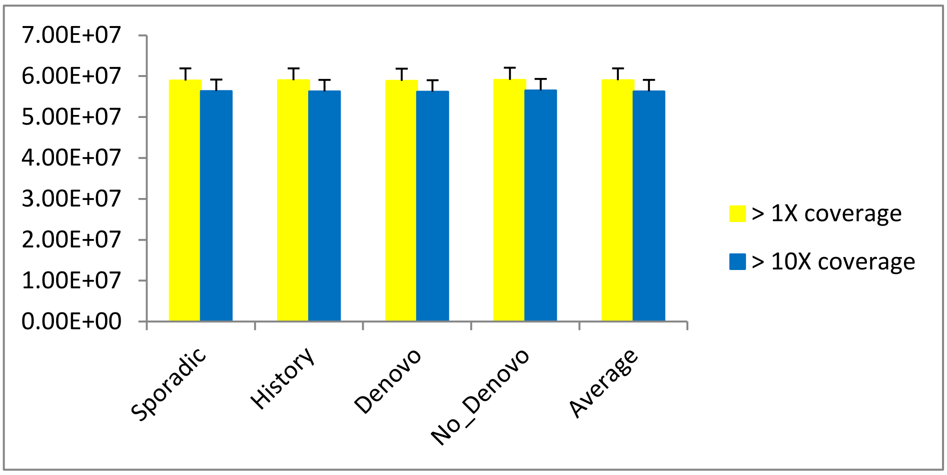

Figure S 2: Sequencing Coverage in Different Groups.

Comparison of average sequence reads at > 1X, and > 10X coverage amongst different sub-groups. There were no significant differences amongst trios with sporadic case and family history, and those with or without *de novo* SNVs.

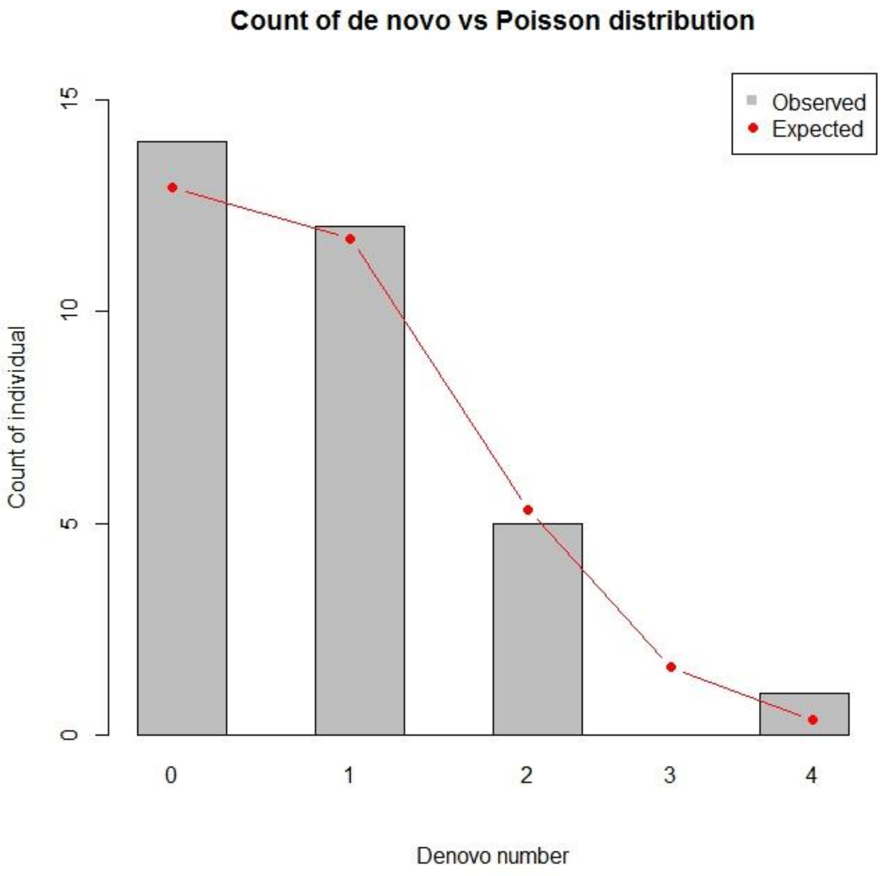

Figure S 3: Density plot of Poisson distribution for observed and expected *de novo* mutations

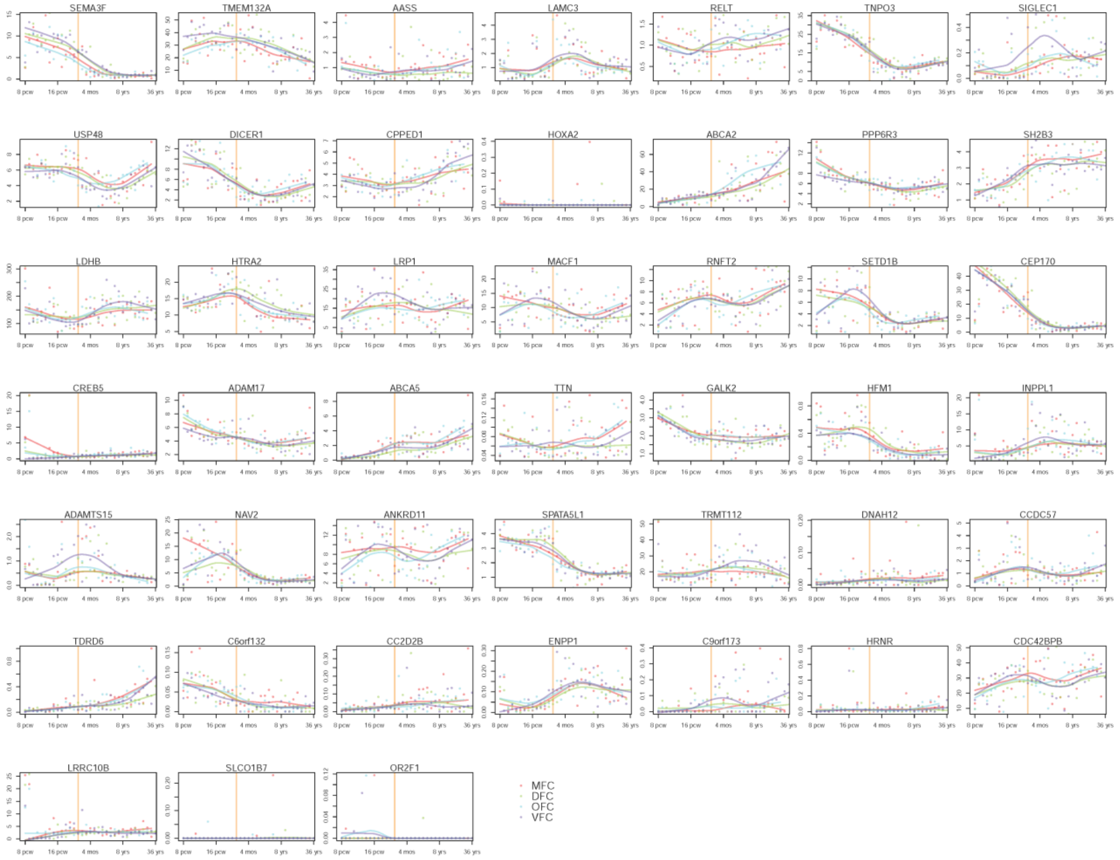

Figure S 4: Expression profiles of genes with damaging DNVs in fetal frontal cortex in different stages

Note: MFC: medial prefrontal cortex, DFC: dorsolateral prefrontal cortex OFC: orbital frontal cortex; VFC: ventrolateral prefrontal cortex.

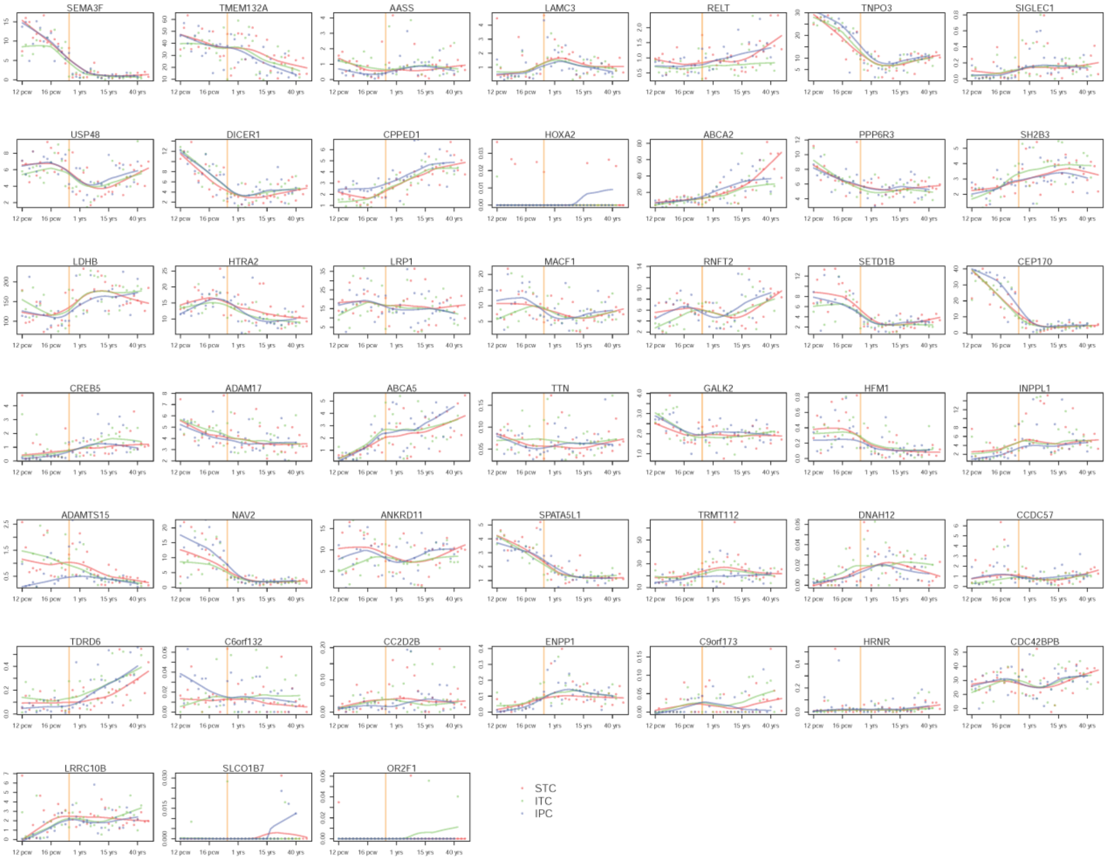

Figure S 5: The expression profiles of genes with damaging DNVs in fetal temporal cortex in different stages

Note: Consistent with the finding of Carlo Colantuoni et al[35,36], we found that the expression changing rate of most genes during fetal development is the fastest in whole human life. During infancy it is much slower, yet still faster than at any later stage in life. After the first half-year of postnatal life, the gene expression changing rate slow down remarkably, and continue to be slow during the childhood and teenage years, and maintain a lower rate through the 20s-40s. After this period, the gene expression changing rates begin to rise again through several decades. In the aged human brain, change reaches and then exceeds the gene expression changing rate observed during teenage years. The profile of expression implicates that the module of neurodevelopment deficit which is consistent with the hypothesis of neurodevelopment deficit of schizophrenia[37]. (The database of BrainSpan do not include the expression data of gene *GTDC2* and *MAATS1*). STC: superior temporal cortex; ITC: Inferolateral temporal cortex; IPC: posteroinferior (ventral) parietal cortex.

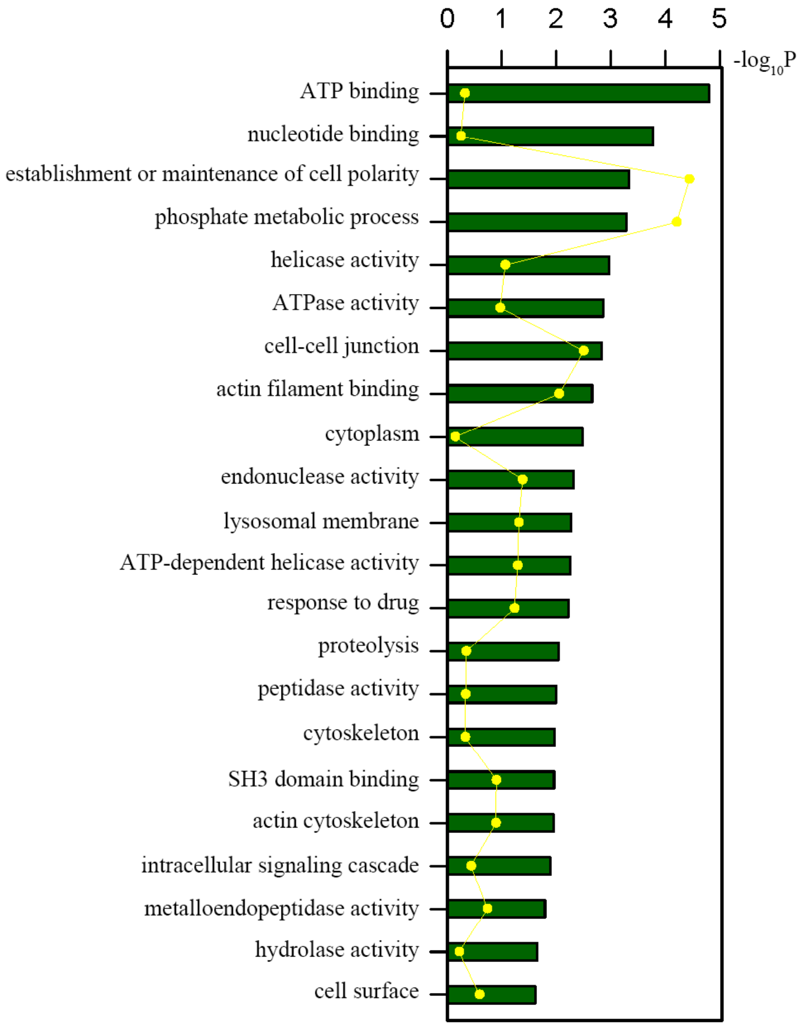

Figure S 6: Pathway analysis of genes carrying *de novo* damaging mutations.

Note: The *p* value for each pathway is indicated by the green bar and is expressed as -1 times the log of the p value. The yellow line and point represents that, in a given pathway, the ratio of the number of genes that meet the cutoff criteria divided by the total number of genes that makes up the pathway (50 times the number of genes carrying *de novo* damaging mutations in each pathway/ the total number of genes in a given pathway). Top 8 GOs remain significance statistically after Bonferroni correction ($p < 0.05$).

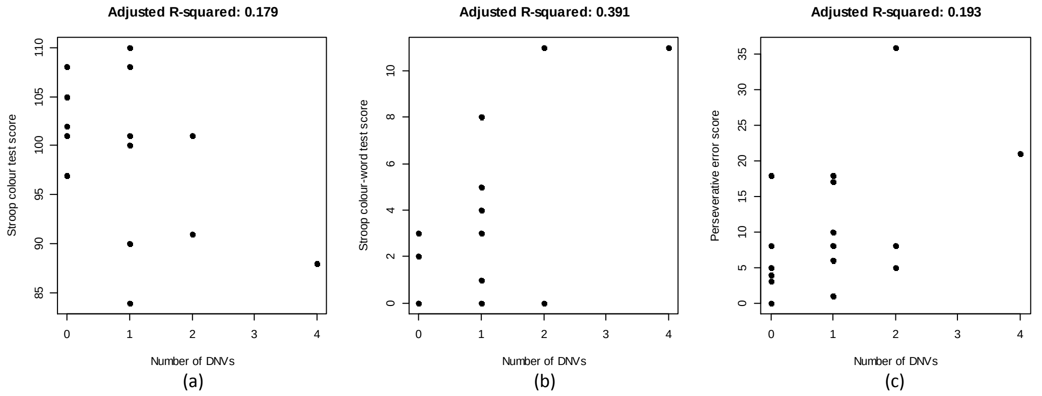

Figure S 7: The scatter plot of total number of DNVs and three cognitive measurements.

Note: Each dot denotes the total number of DNVs and a cognitive measurement of each subject. The subjects with missing value for a cognitive measurement were ignored.

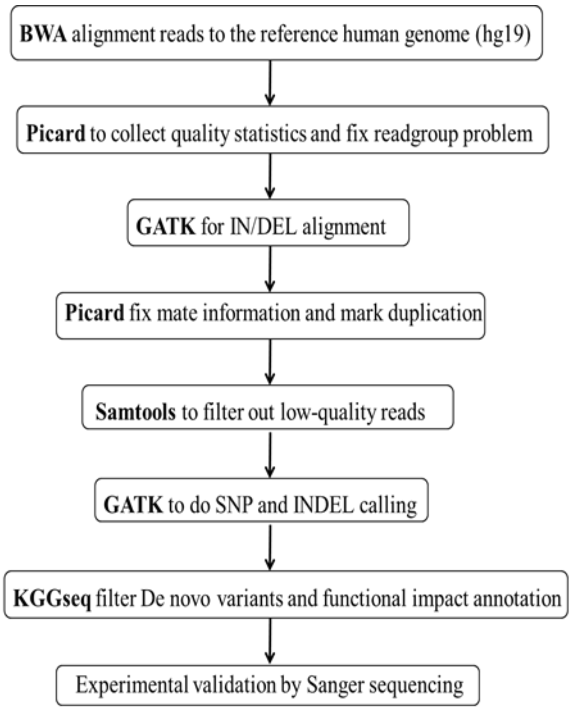

Figure S 8 Sequencing Data Analysis Pipeline

Note: The pipeline describes the procedure for sequence data analysis and *de novo* determination, which includes: (1) BWA alignment reads of raw data to the reference human genome （hg19）; (2) Using Picard to collect quality statistics and fix readgroup problem; (3) GATK for IN/DEL alignment by using dbsnp132 and 1000indel as known IN/DEL sites; (4) Fix mate information and mark duplication with Picard; (5) Using Samtools to filter out low-quality reads or GATK clipping reads of low quality; (6) Using GATK to perform SNP and INDEL calling; (7) KGGseq filter *De novo* variants and functional impact annotation; (8) Experimental validation by Sanger sequencing.

Supplementary Reference

1. Kay, S., Fiszbein, A. & Opler, L. The positive and negative syndrome scale (PANSS) for schizophrenia. *Schizophrenia Bulletin*, 261–276 (1987).
2. Hall, R. Global assessment of functioning. A modified scale. *Psychosomatics* **36**, 267-275 (1995).
3. Groth-Marnat, G. *Handbook of psychological assessment (3rd ed.).* (New York, 1997).
4. Gong, Y.x. *Wechsler Adult Intelligence Scale – Revised in China.*, (Hunan Medical School Press, Hunan, 1982).
5. Gong, Y.x. *Wechsler Memory Scale – Revised in China*, (Hunan Medical School Press., Hunan, 1987).
6. Lezak, M.D., Howieson, D.B. & Loring, D.W. *Neuropsychological assessment (4th ed.).* (Oxford University Press, New York, 2004).
7. Lu, L. & Bigler, E.D. Performance on original and a Chinese version of Trail Making Test Part B: a normative bilingual sample. *Appl Neuropsychol* **7**, 243-246 (2000).
8. Chen, Y.L., Chen, Y.H. & Lieh-Mak, F. Semantic verbal fluency deficit as a familial trait marker in schizophrenia. *Psychiatry Res* **95**, 133-148 (2000).
9. Nelson, H.E. A modified card sorting test sensitive to frontal lobe defects. *Cortex* **12**, 313-324 (1976).
10. Welsh, M.C., Satterlee-Cartmell, T. & Stine, M. Towers of Hanoi and London: contribution of working memory and inhibition to performance. *Brain Cogn* **41**, 231-242 (1999).
11. Spreen, O. & & Strauss, E. *A compendium of neuropsychological tests: Administration,norms, and commentary.* , (Oxford University Press., New York, 1991).
12. Benzel, I. *et al.* Interactions among genes in the ErbB-Neuregulin signalling network are associated with increased susceptibility to schizophrenia. *Behav Brain Funct* **3**, 31 (2007).
13. Wang, Q. *et al.* Normative data on a battery of neuropsychological tests in the Han Chinese population. *J Neuropsychol* **5**, 126-142 (2011).
14. Chan, R.C., Hoosain, R. & Lee, T.M. Reliability and validity of the Cantonese version of the Test of Everyday Attention among normal Hong Kong Chinese: a preliminary report. *Clin Rehabil* **16**, 900-909 (2002).
15. Chan, R.C. & Manly, T. The application of "dysexecutive syndrome" measures across cultures: performance and checklist assessment in neurologically healthy and traumatically brain-injured Hong Kong Chinese volunteers. *J Int Neuropsychol Soc* **8**, 771-780 (2002).
16. Li, M.X., Gui, H.S., Kwan, J.S., Bao, S.Y. & Sham, P.C. A comprehensive framework for prioritizing variants in exome sequencing studies of Mendelian

diseases. *Nucleic Acids Res* **40**, e53 (2012).
17. Genomes Project, C. *et al.* An integrated map of genetic variation from 1,092 human genomes. *Nature* **491**, 56-65 (2012).
18. Kumar, P., Henikoff, S. & Ng, P.C. Predicting the effects of coding non-synonymous variants on protein function using the SIFT algorithm. *Nat Protoc* **4**, 1073-1081 (2009).
19. Adzhubei, I.A. *et al.* A method and server for predicting damaging missense mutations. *Nat Methods* **7**, 248-249 (2010).
20. Chun, S. & Fay, J.C. Identification of deleterious mutations within three human genomes. *Genome Res* **19**, 1553-1561 (2009).
21. Schwarz, J.M., Rodelsperger, C., Schuelke, M. & Seelow, D. MutationTaster evaluates disease-causing potential of sequence alterations. *Nature methods* **7**, 575-576 (2010).
22. Schwarz, J.M., Rodelsperger, C., Schuelke, M. & Seelow, D. MutationTaster evaluates disease-causing potential of sequence alterations. *Nat Methods* **7**, 575-576 (2010).
23. Liu, X., Jian, X. & Boerwinkle, E. dbNSFP v2.0: a database of human non-synonymous SNVs and their functional predictions and annotations. *Hum Mutat* **34**, E2393-2402 (2013).
24. Huang, N., Lee, I., Marcotte, E.M. & Hurles, M.E. Characterising and predicting haploinsufficiency in the human genome. *PLoS genetics* **6**, e1001154 (2010).
25. MacArthur, D.G. *et al.* Guidelines for investigating causality of sequence variants in human disease. *Nature* **508**, 469-476 (2014).
26. Petrovski, S., Wang, Q., Heinzen, E.L., Allen, A.S. & Goldstein, D.B. Genic intolerance to functional variation and the interpretation of personal genomes. *PLoS Genet* **9**, e1003709 (2013).
27. Gratten, J., Visscher, P.M., Mowry, B.J. & Wray, N.R. Interpreting the role of de novo protein-coding mutations in neuropsychiatric disease. *Nat Genet* **45**, 234-238 (2013).
28. Uddin, M. *et al.* Brain-expressed exons under purifying selection are enriched for de novo mutations in autism spectrum disorder. *Nat Genet* (2014).
29. Rimm, D.L., Koslov, E.R., Kebriaei, P., Cianci, C.D. & Morrow, J.S. Alpha 1 (E)-catenin is an actin-binding and-bundling protein mediating the attachment of F-actin to the membrane adhesion complex. *Proceedings of the National Academy of Sciences* **92**, 8813-8817 (1995).
30. Fromer, M. *et al.* De novo mutations in schizophrenia implicate synaptic networks. *Nature* (2014).
31. Hotulainen, P. & Hoogenraad, C.C. Actin in dendritic spines: connecting dynamics to function. *The Journal of cell biology* **189**, 619-629 (2010).
32. Ide, M. & Lewis, D.A. Altered cortical CDC42 signaling pathways in schizophrenia: implications for dendritic spine deficits. *Biological psychiatry* **68**, 25-32 (2010).
33. Enomoto, A. *et al.* Roles of disrupted-in-schizophrenia 1-interacting protein girdin in postnatal development of the dentate gyrus. *Neuron* **63**, 774-787 (2009).

34. Camargo, L. *et al.* Disrupted in Schizophrenia 1 Interactome: evidence for the close connectivity of risk genes and a potential synaptic basis for schizophrenia. *Molecular psychiatry* **12**, 74-86 (2006).
35. Colantuoni, C. *et al.* Temporal dynamics and genetic control of transcription in the human prefrontal cortex. *Nature* **478**, 519-523 (2011).
36. Kang, H.J. *et al.* Spatio-temporal transcriptome of the human brain. *Nature* **478**, 483-489 (2011).
37. Ben-David, E. & Shifman, S. Combined analysis of exome sequencing points toward a major role for transcription regulation during brain development in autism. *Mol Psychiatry* **18**, 1054-1056 (2013).
